# Self-compassion and burnout among medical students in Egypt: indirect effects through perceived stress

**DOI:** 10.1186/s40359-026-04550-1

**Published:** 2026-04-28

**Authors:** Yusof Mohamed Omar, Mariam Wael, Lina Mohamed Omar, Nourhan Amr Elsaid, Naglaa Ahmed El-Imam, Raghad Ismael, Ahmed Abdelmageed, MSSA-Mansoura Team of Collaborators, Renad R. Elbeyaly, Sereen M. Al Tayara, Rofida Mokbel Ibrahim, Khaled Sameh Elbahnasi, HASSAN M. KARRAR, Ahmed A. Shousha, Fatma M. Abu Elsoud, Hassan Moustapha Sabbah, Rodina S. Elkhouly, Rady Turky Amhimd, Mariam M. Baz, Abdel-Hady El-Gilany

**Affiliations:** 1 Faculty of Medicine, Mansoura University, Mansoura, Egypt; 2 Public Health and Community Medicine Department, Faculty of Medicine, Mansoura University, Mansoura, Egypt

**Keywords:** Self-compassion, Stress, Burnout, Medical students, Egypt

## Abstract

**Background:**

Studies among medical students in Egypt consistently report high levels of stress, burnout, and significant hesitancy to seek mental health support. This study investigated the protective role of self-compassion, an internal psychological resource, against burnout.

**Methods:**

A cross-sectional study was conducted using an online questionnaire. A total of 649 medical students from a public university in Egypt were recruited via convenience sampling. Participants completed the Self-Compassion Scale–Short Form (SCS-SF), the Perceived Stress Scale (PSS-10), and the Burnout Assessment Tool (BAT-23). Bivariate associations were tested with Pearson correlations; multivariable linear regression assessed predictors of burnout. Indirect effects analysis was performed with bias-corrected bootstrapped confidence intervals (10,000 samples) to estimate indirect effects.

**Results:**

A total of 649 medical students participated (mean age of 20.6; 58.4% female). Bivariate analyses revealed significant negative correlations between self-compassion and both perceived stress (*r* = -0.577) and burnout (*r* = -0.478), and a positive correlation between stress and burnout (*r* = 0.603; all *p*-values < .001). The multivarivable regression model explained 39.9% of burnout variance (*R*^2^ = .399); perceived stress was the a significant positive predictor (β = 0.488), while self-compassion remained a significant protective factor (β = -0.196). The indirect effects analysis confirmed a significant indirect effect of self-compassion on burnout via perceived stress (B = -0.026, 95% CI [-0.032, -0.021]). As the direct effect also remained significant (B = -0.018), a partial indirect effects model was supported, with the indirect pathway accounting for 59.2% of the total effect.

**Conclusions:**

Higher self-compassion is associated with lower burnout largely via an association with reduced perceived stress. Training self-compassion alongside stress-reduction strategies may help safeguard medical student well-being.

**Supplementary Information:**

The online version contains supplementary material available at 10.1186/s40359-026-04550-1.

## Introduction

Due to the rigorous and demanding nature of medical education, medical students often experience significant stress as they strive to develop the knowledge and skills required to provide patient care. This chronic stress is a contributes significantly to the development of burnout, a syndrome characterized by emotional and cognitive exhaustion, a cynical and detached attitude towards studies, and pervasive feelings of incompetence [1]. The prevalence of burnout among medical students is alarmingly high, with rates estimated at 37.2% globally [2] and as high as 88% in Egypt [3]. Burnout is considered one of the systemic problems in medicine given its associated severe long-term consequences, including poor academic performance, increased rates of anxiety and depression, and diminished empathy, all of which can compromise the quality of future patient care [4].

In response to the widespread prevalence and significant consequences of burnout, researchers have put significant effort into identifying psychological resources that can buffer against its impact. One such resource is self-compassion, which entails responding to personal struggles with kindness rather than self-criticism and maintaining a balanced, mindful perspective on distressing experiences [5]. Research links self-compassion to greater emotional resilience and more effective coping, enabling individuals to face challenges without excessive self-judgment or emotional distress [6]. Furthermore, a growing body of evidence suggests that higher levels of self-compassion are associated with greater psychological well-being, including lower rates of burnout, making it a vital resource for navigating the high-stress environment of medical school [7].

Nevertheless, while research has consistently reported a beneficial relationship between higher self-compassion and lower burnout [8–10], the exact underlying mechanism explaining this relationship remains poorly understood. One explanation is that self-compassion may buffer against burnout by shaping how students appraise and cope with stressors [11]. This notion aligns with Lazarus and Folkman’s transactional model of stress and coping, which entails that stress results from both external demands as well as an individual’s appraisal of those demands [12]. From this perspective, self-compassion may influence both how students interpret the challenges they face, and their capability of coping with them, thereby lowering perceived stress. By responding to academic and psychological challenges associated with studying medicine with self-kindness rather than self-criticism, students may reframe stressors as challenges rather than threats, reducing their likelihood of experiencing chronic stress and, consequently, burnout [13]. Despite this plausible pathway, few studies have empirically examined indirect effects models linking self-compassion, perceived stress, and burnout in the high-stakes context of medical education [14, 15].

With studies among medical students in Egypt consistently reporting high levels of mental distress [16–18], burnout [3], and significant hesitancy to seek mental health support [19], identifying accessible, internal resources for tackling the stress associated with medical education is therefore crucial. However, little research has examined how self-compassion exerts its protective effect against burnout in this population. This study aimed to determine whether perceived stress accounts for the relationship between self-compassion and burnout among medical students through indirect pathways.

## Methods

### Study design and setting

This cross-sectional analytical study was conducted at the Faculty of Medicine, Mansoura University. Data were collected in May 2025. This period was selected as it represents a time in the second academic term when students are sufficiently engaged with the demands and stresses of medical education, yet not subjected to the heightened stress typically associated with final examinations (typically held in June and July). This study followed the strengthening the reporting of observational studies in epidemiology (STROBE) guidelines [20].

### Participants and procedure

The study population comprised all undergraduate students from the first through fifth academic years at the Faculty of Medicine, Mansoura University. Interns and postgraduate students were excluded from participation.

Participants were recruited using a non-random convenience sampling method with proportional allocation across academic years to reflect the student distribution. Data were collected via an anonymous, self-administered online questionnaire created with Google Forms. The survey link was disseminated through official student Telegram channels for each academic year and by student collaborators among their peers.

The introductory page of the survey provided a clear explanation of the study’s aim and guaranteed participant anonymity. Participation was entirely voluntary, and completion of the questionnaire was considered implied informed consent. To encourage participation, respondents were offered the opportunity to view their individual scores for self-compassion, stress, and burnout upon survey completion.

### Sample size

An a-priori power analysis was conducted using the Monte Carlo Power Analysis for Indirect Effects web application [21]. This tool uses a simulation-based approach to estimate the power to detect a hypothesized indirect effect by generating confidence intervals using the Monte Carlo method [22]. For our analysis, we specified a target power of 0.80 at a 95% confidence level. The simulation was based on 1,000 replications and 20,000 Monte Carlo draws per replication. The model was populated with conservative correlation estimates from prior research in a medical student population: −0.21 between self-compassion and stress, 0.52 between stress and burnout, and −0.40 between self-compassion and burnout [15]. The analysis indicated that a minimum sample size of 225 participants was required. To ensure the study was robustly powered to detect a more conservative effect size than suggested by prior research and to enhance the potential generalizability of the findings, we recruited a final sample of 646 participants.

### Measures

We assessed perceived stress using the 10-item Perceived Stress Scale (PSS-10) [23], a widely used self-report measure of individuals’ perception of stress in their lives. Each item is rated on a 5-point Likert scale (0 = never to 4 = very often), yielding a total score ranging from 0 to 40, with higher scores indicating greater perceived stress. We selected the PSS-10 because it captures global stress appraisal, aligning with the Lazarus and Folkman transactional model that underpins our indirect effects hypothesis [12]. Although the scale can be analyzed using its two-factor structure (perceived helplessness and perceived self-efficacy) [23], we used the total score to reflect overall perceived stress, which was most relevant to our indirect effects model. The PSS-10 has been used in a previous study among medical students in Egypt [24], and has shown good reliability in a study in a similar population of university students in Saudi Arabia [25]. In the present sample, the PSS-10 demonstrated acceptable reliability (Cronbach’s α = 0.796, McDonald’s ω = 0.800). Confirmatory factor analysis (CFA) of the unidimensional model showed suboptimal fit (Comparative Fit Index (CFI) = 0.740, Root Mean Square Error of Approximation (RMSEA) = 0.149); however, this pattern is consistent with the well-documented method effect of the scale’s four reversed items, which introduce a spurious secondary factor unrelated to substantive differences in the stress construct [26]. Exploratory factor analysis (EFA) confirmed the established two-factor structure of perceived helplessness and perceived self-efficacy; consistent with prior literature and the original developers’ recommendation [23, 27], the total score was retained as a unidimensional measure of global stress appraisal.

To measure the participants’ self-compassion, we used the 12-item Self-Compassion Scale – Short Form (SCS-SF) [28], a validated alternative to the original 26-item SCS that reliably measures the single higher-order factor of overall self-compassion. Each item is rated on a 5-point Likert scale (1 = Almost Never to 5 = Almost Always), with total scores ranging from 12 to 60; higher scores reflect greater self-compassion. Given the legnth of the survey and to minimize potential response fatigue, we used the SCS-SF as a unifactorial measure by calculating a total score. The construct validity of the original SCS has been supported in a study with Egyptian and Saudi university students, confirming the relevance of the self-compassion construct in this population [29]. In the present sample, the SCS-SF demonstrated acceptable reliability (Cronbach’s α = 0.764, McDonald’s ω = 0.766). CFA of the unidimensional model showed suboptimal fit (CFI = 0.636, RMSEA = 0.128). EFA revealed a dominant first eigenvalue with a steep scree drop, supporting sufficient unidimensionality for total score use. As recommended by the scale developers and consistent with its design as a measure of overall self-compassion [28], only the total score was used in analyses.

Burnout was measured using the 23-item Burnout Assessment Tool – Student version (BAT-23), a recently validated instrument demonstrating strong convergent and discriminant validity with other burnout scales, including the Maslach and Oldenburg Burnout Inventories [1, 30]. The BAT-23 is based on Schaufeli et al.’s four-dimensional model of burnout, encompassing exhaustion, mental distancing, cognitive impairment, and emotional impairment. Items are rated on a 5-point Likert scale (1 = never, 5 = always), with higher scores indicating greater burnout. The BAT-23 has demonstrated excellent reliability in a recent study among Egyptian medical students, which reported a Cronbach’s alpha of 0.92 consistent with the reliability reported in the original validation study [1].

Lastly, we collected non-identifying participant sociodemographic data, including age (in years), sex, nationality, residence (urban vs rural), and academic year. Additionally, students were asked to self-report whether they’ve been previously diagnosed with a mental health disorder.

### Data analysis

All statistical analyses were performed using Jamovi (version 2.3.28). Continuous variables were summarized as means and standard deviations (SDs) and assessed for normality via visual inspection of Quantile–Quantile (Q-Q) plots, while categorical variables were reported as frequencies and percentages. To assess the factorial validity of the study measures in the present sample, CFA was conducted for each scale. Where CFA fit was suboptimal, EFA was conducted as a supplementary check.

Due to a data collection error, one item from the BAT-23 was inadvertently omitted from the online questionnaire, and the item was thus never presented to affected respondents rather than being skipped, resulting in missing data for 224 participants. We addressed this using person-mean substitution, imputing the missing value with the mean of the participant’s responses to the remaining items within the same BAT subscale. This approach was chosen to preserve statistical power and was supported by the high inter-item correlations within the BAT subscales and the negligible impact on scale reliability (e.g., Cronbach’s α for total BAT score: 0.921 post-imputation vs. 0.919 pre-imputation).

Group differences in mean scores were assessed using independent-samples t-tests for sociodemographics with two groups and one-way ANOVA for differences between academic years, followed by Tukey’s post hoc tests for pairwise comparisons when ANOVA results were significant. Pearson’s correlation was used to examine the relationships between self-compassion, stress, and burnout. Linear regression was conducted to identify predictors of burnout.

To test our primary hypothesis regarding indirect effects, we conducted an indirect effects analysis using the medmod module in Jamovi, with self-compassion as the independent variable, burnout as the dependent variable, and perceived stress as the mediator. The significance of the indirect effect was evaluated using bootstrapped 95% confidence intervals based on 10,000 samples. To address the limitations of cross-sectional indirect effects analysis, alternative directional models were also tested, without bootstapping, to determine if the data supported competing theoretical pathways. For all analyses, statistical significance was set at *p* < 0.05.

## Results

### Participant characteristics and bivariate associations

The study included 649 medical students with a mean age of 20.6 (SD = 1.87) years. The majority of participants were female (58.4%) and of Egyptian nationality (76.9%). The mean scores were 36.4 (SD = 6.75) for self-compassion, 22.2 (SD = 5.89) for perceived stress, and 3.38 (SD = 0.63) for burnout (Table 1).

**Table 1:** Participant characteristics and comparison of self-compassion, stress, and burnout scores (*N* = 649)

Characteristic		n (%)	Self-compassion^1,2^	*p*	Stress^1,3^	*p*	Burnout^1,4^	*p*
Overall			36.4 (6.75)		22.2 (5.89)		3.38 (0.63)	
Age, years^1^		20.6 (1.87)						
Sex	Female	395 (58.4%)	36.21 (7.05)		23.58 (5.75)		3.46 (0.63)	
	Male	270 (41.6%)	36.76 (6.30)	0.309	20.15 (5.49)	**<.001**	3.26 (0.61)	**<.001**
Nationality	Egyptian	499 (76.9%)	36.04 (6.72)	**0.006**	22.87 (5.73)	**<.001**	3.42 (0.64)	**0.002**
	Non Egyptian	150 (23.1%)	37.75 (6.72)		19.77 (5.78)		3.24 (0.57)	
Residence	Rural	254 (39.1%)	36.50 (5.88)	0.837	22.01 (5.42)	0.612	3.41 (0.65)	0.318
	Urban	395 (60.9%)	36.39 (7.27)		22.25 (6.18)		3.36 (0.62)	
Academic year	1 st year	153 (23.6%)	36.99 (6.43)	0.137	20.87 (6.02)^a,b^	**<.001**	3.23 (0.59)^a,b^	**0.003**
	2nd year	148 (22.8%)	35.72 (7.18)		23.30 (6.29)		3.47 (0.66)	
	3rd year	111 (17.1%)	37.23 (6.94)		20.98 (6.12)^a,b^		3.31 (0.62)	
	4th year	134 (20.6%)	35.54 (6.79)		23.23 (5.15)		3.48 (0.63)	
	5th year	103 (15.9%)	36.96 (6.20)		22.28 (5.19)		3.40 (0.61)	
Mental health disorder	No	518 (79.8%)	36.92 (6.65)	**<.001**	21.84 (5.82)	**0.006**	3.35 (0.62)	**0.002**
	Yes	131 (20.2%)	34.53 (6.85)		23.41 (6.01)		3.48 (0.67)	

^1^Mean (SD)

^2^Self-Compassion Scale–Short Form (SCS-SF), possible range: 12–60

^3^Perceived Stress Scale (PSS-10), possible range: 0–40

^4^Burnout Assessment Tool–Student version (BAT-23), possible range: 23–115

^a^Significantly less than 2nd years

^b^Significantly less than 4th years

Bold values indicate statistically significant results (*p* < 0.05)

Female students reported higher stress (23.58 vs. 20.15; *p* < 0.001) and burnout (3.46 vs. 3.26; *p* < 0.001) compared to male students. A similar pattern was observed among Egyptian students and those with a self-reported mental health disorder, who consistently reported significantly higher stress and burnout, and lower self-compassion, compared to their counterparts (*p* < 0.05). Significant differences were also found across academic years, with first-year students generally experiencing lower stress and burnout compared to those in their second and fourth years (Table 1).

Consistent with these patterns, self-compassion correlated negatively with perceived stress (*r* = − 0.577, *p* < 0.001) and burnout (*r* = − 0.478, *p* < 0.001), while perceived stress correlated positively with burnout (*r* = 0.603, *p* < 0.001). In terms of burnout subscales, self-compassion was most negatively correlated with the affective and energetic dimensions of burnout, namely emotional impairment (*r* = − 0.484, *p* < 0.001) followed by exhaustion (*r* = − 0.375, *p* < 0.001) (Table 2).

**Table 2:** Pearson correlation matrix of participants’ self-compassion, perceived stress, and burnout scores table

Variable	1	2	3	4	5	6	7
1. Total Burnout	—						
2. Exhaustion	0.810^***^	—					
3. Mental Distancing	0.748^***^	0.501^***^	—				
4. Cognitive Impairment	0.850^***^	0.618^***^	0.517^***^	—			
5. Emotional Impairment	0.784^***^	0.488^***^	0.434^***^	0.548^***^	—		
6. Stress	0.603^***^	0.542^***^	0.467^***^	0.577^***^	0.484^***^	—	
7. Self-Compassion	−0.478^***^	−0.375^***^	−0.280^***^	−0.374^***^	−0.484^***^	−0.577^***^	—

^***^p <.001

### Factors associated with burnout

The overall regression model was statistically significant, explaining 39.9% of the variance in total burnout scores (*R*^2^ = 0.399) (Table 3). Perceived stress was a significant positive predictor of burnout (*β* = 0.49, *p* < 0.001), while self-compassion was a significant negative predictor (*β* = −0.20, *p* < 0.001). Furthermore, compared to first-year students, being in the second, fourth, or fifth academic year was associated with higher burnout (*p* < 0.05). Additionally, students from a rural residence reported significantly higher burnout than those from urban areas (*β* = 0.128, *p* = 0.048).

**Table 3:** Multiple linear regression of factors associated with burnout among medical students

Variables	B [95% CI]	β [95% CI]	*p*
Age (continuous)	−0.03 [−0.06, 0.01]	−0.09 [−0.19, 0.02]	0.11
Sex (Ref: Female)			
Male	0.02 [−0.07, 0.11]	0.03 [−0.12, 0.17]	0.693
Nationality (Ref: Egyptian)			
Non-Egyptian	0.05 [−0.05, 0.16]	0.08 [−0.08, 0.25]	0.319
Residence (Ref: Urban)			
Rural	0.08 [0.00, 0.16]	0.13 [0.00, 0.26]	**0.048**
Mental Health Disorder (Ref: No)			
Yes	0.02 [−0.08, 0.11]	0.03 [−0.13, 0.18]	0.719
Academic Year (Ref: 1 st Year)			
2nd Year	0.14 [0.01, 0.26]	0.22 [0.02, 0.42]	**0.034**
3rd Year	0.16 [−0.00, 0.32]	0.25 [−0.01, 0.50]	0.057
4th Year	0.20 [0.03, 0.37]	0.32 [0.05, 0.59]	**0.02**
5th Year	0.25 [0.04, 0.45]	0.39 [0.07, 0.71]	**0.017**
Perceived Stress	0.05 [0.04, 0.06]	0.49 [0.41, 0.57]	**<.001**
Self-compassion	−0.02 [−0.03, −0.01]	−0.20 [−0.27, −0.12]	**<.001**

*B* unstandardized coefficient, *β* standardized coefficient, *CI* Confidence Interval

Bold values indicate statistically significant results (*p* < 0.05)

As detailed in Table 3, after controlling for all other variables, perceived stress was a significant positive predictor of burnout (β = 0.49, *p* < 0.001). Self-compassion was a significant negative predictor, with higher levels associated with lower burnout scores (β = −0.20, *p* < 0.001). Furthermore, compared to first-year students, being in the second (B = 0.14, *p* = 0.034), fourth (B = 0.20, *p* = 0.020), or fifth academic year (B = 0.25, *p* = 0.017) was associated with higher burnout. Additionally, students from a rural residence reported significantly higher burnout than those from urban areas (B = 0.08, *p* = 0.048).

### Indirect role of perceived stress

To test the primary hypothesis that perceived stress statistically accounts for the relationship between self-compassion and burnout, an indirect effects analysis was conducted. The results, detailed in Table 4, supported a partial indirect effects model.

**Table 4:** Analysis of the indirect effect of self-compassion on burnout through perceived stress

Pathway	*B*	95% CI	β	95% CI	*p*
Model Paths					
Path a: Self-compassion → Stress	− 0.504	[− 0.564, − 0.442]	− 0.577	[− 0.647, − 0.507]	<.001
Path b: Stress → Burnout	0.052	[0.045, 0.060]	0.490	[0.419, 0.563]	<.001
Summary Effects					
Indirect (a × b)	− 0.026	[− 0.032, − 0.021]	− 0.283	[− 0.340, − 0.231]	<.001
Direct (c)	− 0.018	[− 0.025, − 0.012]	− 0.195	[− 0.265, − 0.123]	<.001
Total (c + ab)	− 0.045	[− 0.050, − 0.039]	− 0.478	[− 0.542, − 0.416]	<.001

*B* unstandardized coefficient, *β* standardized coefficient, *CI* confidence interval

Bold values indicate statistically significant results (*p* < 0.05)

Initially, the total effect of self-compassion on burnout was significant (*B* = −0.045, β = − 0.478, *p* < 0.001). In the indirect effects model, higher self-compassion significantly predicted lower perceived stress (Path *a*: *B* = −0.504, β = − 0.577, *p* < 0.001), and higher perceived stress, in turn, predicted higher burnout (Path *b*: *B* = 0.052, β = 0.490, *p* < 0.001).

**Fig. 1:**
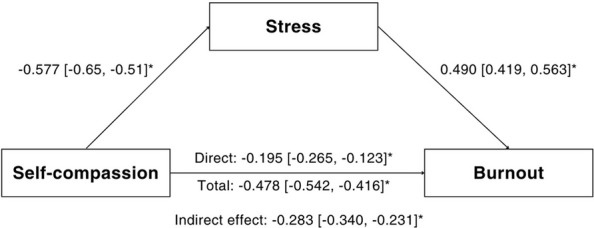
Standardized regression coefficients (β) and 95% confidence intervals for the relationship between self-compassion and burnout with perceived stress as an intermediary. Asterisk indicates significance level: *p <.001

The analysis revealed a significant indirect effect of self-compassion on burnout through stress (*B* = −0.026, β = − 0.283). The direct effect of self-compassion also remained significant (*B* = −0.018, β = − 0.195, *p* < 0.001). The indirect effect accounted for approximately 59.2% of the total effect, suggesting that a substantial portion of the protective relationship between self-compassion and burnout association between self-compassion and burnout is statistically attributable to reduced perceived stress (Fig. 1).

Given the cross-sectional nature of the data, three alternative directional models were tested to evaluate competing pathways (Supplementary Material 1). All alternative models yielded statistically significant indirect effects. Standardized comparisons indicated that the primary model (β = − 0.283, 59.2% mediation) produced a larger indirect effect than Model 2 (stress mediates burnout → self-compassion: β = − 0.274, 57.2%), Model 3 (self-compassion mediates stress → burnout: β = 0.113, 18.7%), and Model 4 (self-compassion mediates burnout → stress: β = 0.179, 29.7%).

## Discussion

In a culture where studies among medical students in Egypt consistently report high levels of stress [16], burnout [3, 31], and significant hesitancy to seek mental health support [19], this study investigated the protective role of self-compassion against burnout. We found that self-compassion correlated negatively with both perceived stress and burnout. Further, indirect effects analysis indicated that a substantial portion of the association was statistically attributable to perceived stress. These findings position self-compassion as a potential target for both institutional and educational interventions aimed at mitigating burnout within this population.

Our finding that perceived stress partially accounts for the relationship between self-compassion and burnout aligns with several recent studies. For instance, Meng et al. similarily found that perceived stress statistically accounted for the relationship between self-compassion and anxiety/depression amongst medical workers [32]. Similarly, Zhao et al. also found that self‑compassion was both directly and indirectly associated with decreased depressive symptoms by increasing resilience and optimism and reducing perceived stress among Chinese medical and nursing students [14]. This pattern is reinforced by longitudinal data, where a cross-lagged panel study among students concluded that perceived stress mediated the link between self-compassion and well-being over time [33]. Our results are further in line with those of Dev et al., who also reported significant relationships between self-compassion, stress, and burnout in healthcare trainees [15]. However, unlike Dev et al., who conceptualized self-compassion as a moderator of the stress–burnout relationship and found no significant effect in medical students, we modeled self-compassion as an antecedent to stress and demonstrated a significant indirect effect on burnout. This suggests that self-compassion’s primary contribution may lie in reducing the experience of stress rather than buffering its impact on burnout.

This interpretation is consistent with Lazarus and Folkman’s transactional model of stress and coping, which posits that stress arises from external demands and an individual’s appraisal of those demands [12]. From this perspective, self-compassion may shape how students view the challenges associated with medical education as well as their coping capacity, thereby reducing perceived stress. In line with this model, our findings showed a substantial indirect effect (≈59% of the total), highlighting stress reduction as a key mechanism. Nevertheless, the indirect effects was partial, as a significant direct effect remained, indicating that self-compassion also influences burnout through additional pathways.

One possible mechanism is that self-compassion has been shown to increase resilience and engagement in self-care behaviors, which can reduce core symptoms of burnout. Using the BAT-23, our analyses showed that self-compassion was negatively correlated with emotional impairment and exhaustion, suggesting it buffers against the emotional and energetic depletion central to severe burnout [14, 34]. This mechanism would be especially perneient to our sample, as studies among medical students in Egypt have repeatedly reported low resilience levels [16, 35], and pessimistic attitude towards studying medicine [36]. Other studies highlight the role of psychological capital (hope, optimism, self-efficacy) and compassion satisfaction as mediators linking self-compassion to lower burnout and depression [14, 37]. Self-compassion has also been associated with greater fulfillment of basic psychological needs for autonomy, competence, and relatedness, which are known protective factors against burnout [38].

Nevertheless, alternative causal sequences than the one reported in our study are both theoretically and empirically plausible. Our supplementary analyses demonstrated that alternative directional models, stress acting as an intermediary between burnout and self-compassion, or self-compassion acting as an intermediary between stress and burnout, were statistically equivalent to our primary model, aligning with existing literature. For instance, Serrão et al. reported an alternative model among Portuguese university students in which stress predicted lower self-compassion, which in turn predicted higher depressive symptoms [39]. Adding further complexity, a 4-year longitudinal study by Park et al. found that an increase in stress actually predicted a subsequent increase in self-compassion, supporting a “stress inoculation” model [40]. Taken together, these findings suggest a reciprocal relationship where self-compassion mitigates the initial experience of stress, while the process of successfully navigating moderate stress may, in turn, build self-compassion over time. Future research should aim to disentangle these pathways through longitudinal and experimental designs that test whether interventions that cultivate self-compassion produce downstream reductions in burnout.

Beyond the primary indirect effects model, our study identified several demographic factors associated with student distress. The finding that female students reported significantly higher stress and burnout is consistent with a large body of research, including a multi-center study of Egyptian medical students [16] and a previous study at our own institution [35], both of which identified female gender as a significant risk factor for lower resilience. One potential explanation for this disparity may be related to barriers to mental health care. Studies on Egyptian students by have repeatedly reported a significant hesitancy to seek help due to factors like social stigma [41], wanting to solve their mental health problems on their own [19], and lack of trust in mental health services [42], barriers which may disproportionately affect female students. Our finding that burnout was significantly higher in later academic years differs from Serrão et al. who found that distress was highest among first-year university students in Portugal [39]. This discrepancy may reflect differences in curricular demands, with later years of Egyptian medical education being more academically and clinically intensive.

Our findings have significant practical implications for medical education and student support services, as self-compassion is not a fixed trait but a skill that can be cultivated [43]. As an internal psychological resource, it represents an especially promising intervention target for Egyptian medical students, who consistently report significant barriers and reluctance to seeking formal mental health care. Our results highlight the value of prioritizing such interventions for vulnerable subgroups, particularly female students, those from rural areas, and those in the more demanding later years of training. Embedding evidence-based programs, such as Mindful Self-Compassion workshops which a recent meta-analysis found to be effective [44], within the curriculum may provide a proactive and scalable strategy to enhance coping, reduce stress, and ultimately prevent burnout among future physicians.

## Limitations

Nevertheless, several limitations should be considered when interpreting the study’s findings. The cross-sectional design precludes causal inference. Although the primary model was theory-driven, supplementary analyses showed that alternative directional models were equally plausible. Accordingly, the findings indicate an indirect association consistent with mediation, but longitudinal studies are needed to determine temporal ordering. The use of convenience sampling at a single institution may limit generalizability, highlighting the need for multi-center studies across diverse Egyptian medical schools to strengthen external validity. Reliance on self-report instruments introduces the possibility of response and recall bias, underscoring the value of incorporating objective or mixed-methods assessments in future work. Furthermore, confirmatory factor analysis of the PSS-10 and SCS-SF revealed suboptimal model fit in the present sample. While EFA and acceptable reliability coefficients supported the use of total scores, findings involving these measures should be interpreted with this caveat in mind. Finally, the systematic omission of one BAT-23 item due to a design error represents a limitation of the imputation approach used; although the negligible impact on scale reliability supports the validity of this approach, future studies should verify data collection instruments prior to deployment to avoid design-level missing data.

## Conclusion

The findings of this study suggest that self-compassion is a vital psychological resource that protects against burnout among Egyptian medical students, with a substantial portion of this association statistically attributable to mitigating perceived stress. These results suggest that interventions aimed at cultivating self-compassion may be a practical strategy for medical educators and student wellness programs, especially in resource constrained settings such as Egypt. Fostering the ability of future physicians to respond to training challenges with self-kindness is a critical step in reducing burnout and building a sustainable, resilient healthcare workforce.

## Supplementary Information

Supplementary Material 1.

## Data Availability

The data of this study is available from the corresponding author upon reasonable request.

## Abbreviations

BAT-23: Burnout Assessment Tool
CFI: Comparative Fit Index
CI: Confidence Interval
CFA: Confirmatory Factor Analysis
EFA: Exploratory Factor Analysis
IRB: Institutional Research Board
PSS-10: Perceived Stress Scale
Q-Q: Quantile-Quantile
RMSEA: Root Mean Square Error of Approximation
SCS: Self-Compassion Scale
SCS-SF: Self-Compassion Scale–Short Form
SD: Standard Deviation
STROBE: Strengthening the Reporting of Observational Studies in Epidemiology
