# Supplementary material for: Self-compassion and burnout among medical students in Egypt: indirect effects through perceived stress
Source: BMC Psychol. 2026 Apr 28;14:621. doi: 10.1186/s40359-026-04550-1 (PMC13126970; doi:10.1186/s40359-026-04550-1)
Supplement: Supplementary file 1 — Supplementary Material 1. [file 40359_2026_4550_MOESM1_ESM.docx]

**Supplementary Table 1: Comparison of Four Standardized Indirect Effects Models**

|  | **Model 1 Stress as intermediary: SC → Burnout** | **Model 2 SC as intermediary: Stress → Burnout** | **Model 3 Stress as intermediary: Burnout → SC** | **Model 4 SC as intermediary: Burnout → Stress** |
| --- | --- | --- | --- | --- |
| **Path a (Predictor → Mediator)** | | | | |
| **β (Estimate)** | −0.577 | −0.577 | 0.603 | −0.478 |
| **SE** | 0.0321 | 0.0321 | 0.0313 | 0.0345 |
| **95% CI** | [−0.640, −0.515] | [−0.640, −0.515] | [0.541, 0.664] | [−0.546, −0.411] |
| **p** | < .001 | < .001 | < .001 | < .001 |
| **Path b (Mediator → Outcome)** | | | | |
| **β (Estimate)** | 0.490 | −0.195 | −0.454 | −0.375 |
| **SE** | 0.0376 | 0.0376 | 0.0394 | 0.0325 |
| **95% CI** | [0.416, 0.564] | [−0.269, −0.122] | [−0.531, −0.377] | [−0.438, −0.311] |
| **p** | < .001 | < .001 | < .001 | < .001 |
| **Direct Effect (c path)** | | | | |
| **β (Estimate)** | −0.195 | 0.490 | −0.205 | 0.424 |
| **SE** | 0.0376 | 0.0376 | 0.0394 | 0.0325 |
| **95% CI** | [−0.269, −0.122] | [0.4165, 0.564] | [−0.282, −0.127] | [0.360, 0.487] |
| **p** | < .001 | < .001 | < .001 | < .001 |
| **Indirect Effect (a × b)** | | | | |
| **β (Estimate)** | −0.283 | 0.113 | −0.274 | 0.179 |
| **SE** | 0.0268 | 0.0226 | 0.0277 | 0.0202 |
| **95% CI** | [−0.335, −0.230] | [0.0685, 0.157] | [−0.328, −0.219] | [0.140, 0.219] |
| **p** | < .001 | < .001 | < .001 | < .001 |
| **Total Effect** | | | | |
| **β (Estimate)** | −0.478 | 0.603 | −0.478 | 0.603 |
| **SE** | 0.0345 | 0.0313 | 0.0345 | 0.0313 |
| **95% CI** | [−0.546, −0.411] | [0.5415, 0.664] | [−0.546, −0.411] | [0.541, 0.664] |
| **p** | < .001 | < .001 | < .001 | < .001 |
| **Indirect Effects Summary** | | | | |
| **% Mediation** | **59.2%** | **18.7%** | **57.2%** | **29.7%** |
| **% Direct** | 40.8% | 81.3% | 42.8% | 70.3% |
| *** p < .001. CI = Confidence Interval. SE = Standard Error. SC = Self-compassion | | | | |
